# GUCY2C at the intersection of obesity and colorectal cancer

**DOI:** 10.1186/2050-6511-14-S1-O28

**Published:** 2013-08-29

**Authors:** Scott A Waldman, Jieru E Lin, Francheska Colon-Gonzalez, Adam Snook, Michael Valentino, Gilbert Kim, Erik Blomain, Theresa Hyslop

**Affiliations:** 1 Department of Pharmacology and Experimental Therapeutics, Thomas Jefferson University, Philadelphia, Pennsylvania, USA

## 

There is an emerging link between body mass and intestinal malignancies, and obese patients have ~20-60% greater risk of, and ~2-fold greater mortality from, colorectal cancer. Although this epidemiological relationship is established, mechanisms that link obesity and colorectal tumorigenesis, and their reversibility, remain incompletely defined. Here, we explore the novel hypothesis that obesity and colorectal cancer are mechanistically linked through dysregulation of paracrine and endocrine hormone axes mediated by GUCY2C. GUCY2C is the receptor for the paracrine hormones guanylin in the colorectum and uroguanylin in small intestine. These paracrine hormones are the most commonly lost gene products in sporadic colorectal cancer, and their universal loss early in neoplasia is required for tumor initiation and progression in mice and humans. In that context, GUCY2C regulates intestinal homeostasis, and its silencing by hormone loss accelerates proliferation and the cell cycle, increases DNA damage, and reprograms cellular metabolism, which increases genetic- and carcinogen-induced colorectal cancer in mice. Beyond its role in colorectal tumor suppression, GUCY2C and uroguanylin comprise a novel gut-brain endocrine axis, evolutionarily conserved from invertebrates to humans, that regulates appetite, body mass and metabolism. Elimination of this endocrine axis in mice results in hyperphagia, obesity, diabetes and the metabolic syndrome. Unexpectedly, recent studies revealed that GUCY2C paracrine hormone expression in colon is eliminated by diet-induced obesity in mice and humans. Indeed, hormone expression appears to be reversibly modulated by ingested calories, rather than by the endocrine, adipokine or inflammatory milieu associated with obesity. Moreover, enforced expression of GUCY2C paracrine hormone by intestinal epithelial cells eliminates tumorigenesis induced by obesity. These observations suggest a model of cancer risk in which ingested calories contributing to obesity recapitulate mechanisms underlying sporadic colorectal cancer by suppressing paracrine hormone expression, silencing the GUCY2C tumor suppressor and disrupting epithelial homeostasis. They define a previously unrecognized mechanism linking diet and obesity to colorectal cancer and identify silencing of the GUCY2C tumor suppressor as a link between reversible risk factors like ingested calories and molecular mechanisms underlying cancer development. Moreover, they offer strategies for countering these risks, including calorie restriction and oral hormone therapy.

